# The Cure of Epithelioma

**Published:** 1892-01

**Authors:** W. S. Gottheil

**Affiliations:** New York


### THE CURE OF EPITHELIOMA.

W. S. GOTTHEIL, M. D., NEW YORK.

The existence of a carcinomatous element being recognized, the question as to the most appropriate treatment for it at once presents itself. And whilst there may be much divergence of opinion as to what this treatment should be, on one point we are all agreed, viz: That it should aim at the complete removal of every particle of epitheliomatous tissue—of every single outlying cell that may have felt the influence of that mysterious stimulus that has awakened it into a life of such pertinaceous activity. For experience teaches the probability of the fact that the leaving behind of a single one of these cells will inevitably lead to the recurrence of the disease.

Hence, we can at once cast aside a number of methods of treatment which have been recommended. All manner of internal medication is absolutely useless. No drug has ever been known, and, so far as we can foresee, no drug will ever be known, the ingestion and assimilation of which has the slightest effect on epitheliomatous tissue. It is true that there are cases recorded to the contrary. Many patients—and alas! also some physicians, tell us of the wonderful results they have seen in the "melting away" of skin cancers under the exhibition of certain remedies. Only recently I received the assurances of an old and esteemed practitioner of the wonderful effects of a well advertised patent medicine in a case of fungating epithelioma. The error is either one of observation, and the growth has not improved during the medication, or the diagnosis is at fault, and the new growth was really a tubercular ulcerative syphilodema. The alleged skin remedy in question undoubtedly contains mercury and iodide of potassium ; hence its action on "syphilitic cancers" is not a matter to be wondered at.

In the same way we can cast aside without examination all local remedies that are anything less than deep escharotics. No matter how early we begin the treatment of our epithelioma, we are already dealing with a dense mass of proliferating epithelioma, the superficial layers of which afford a very efficient protection to the deeper cells against any superficial injuries.

We shall therefore waste no time in painting the tumor with Fowler's solution or applying salicylic acid in solution, or stick of nitrate of silver, or bluestone. These things are not only useless—they are absolutely harmful. They exert whatever slight amount of destructive power they may possess on the superficial layer of the growth only; whilst on the deeper and rapidly growing masses they act as a direct stimulant, exciting them to more rapid and more vigorous growth. They not only do not destroy the cancerous tissue—they directly favor its growth.

There remains then as worthy of our consideration only the more radical and deep-reaching methods. These consist of:

1. The knife.
2. Electrolysis.
3. The sharp curette.
4. Escharotics.

Each one of these methods has its enthusiastic advocates. Each one of them is undoubtedly the best in certain cases. A wise eclectricism is the most rational plan. Let me try and indicate in what class of cases I have obtained the best results from each of them.

1. The patient invariably objects to the knife. Whether this fear is legitimate or not on his part matters little. It is a factor with which we have to deal. Cæteris parilus, he will be most successful in treating his cases who uses the knife least. Nor is the fear of the necessary anæsthetic without weight in the patient's mind. But there is another and more serious objection to the knife, and one which should have more weight with the physician. The mode of growth of the epithelial mass is a peculiar one. The cells grow in nests, which form arms and projections that invade the surrounding tissue. It is only when they have accumulated in large quantities that they begin to form the characteristic pearly elevations that betray the presence of the disease to us. Hence there are always outside the apparent limits of the disease areas of skin that have already been invaded by the cell-nests. Of the extent of these the surgeon has no means of judging. He cannot tell where his incision should be made. However far outside the apparent limits of the neoplasm he may make his mark, he is still liable to cut off and leave behind projecting arms of the epithelioma, which will soon form new centres for malignant growth.

Thus it happens that relapses are more common after excision than after any other radical measure. This, together with the patient's years, form the main objection to this plan of treatment.

Nevertheless, in a certain class of cases it is the most available and perhaps the only plan. This is the case in all cases where the epithelium invades or is in very close proximity to the mucous membrane at one of the natural orifices. Here some of the applications to be hereafter mentioned cannot be used on account of their poisonous nature, and the danger of their absorption; and others again cause so much inflammatory action in their immediate vicinity as to endanger important organs.

So we prefer the knife in all epithelial carcinomata that involve the angle of the mouth, or the lips; in all that impinge upon the orifices of the nostrils, or the upper or lower eyelids. Here excision, as far as we possibly can through the mucous membrane and skin beyond the apparent limits of the disease is our best plan of treatment. Nevertheless, even in these cases it is frequently possible, and always advisable where it is possible, to treat the parts of the tumor furthest removed from the mucous membrane by one or other of the methods to be described below.

Local anæsthesia is rarely sufficient where the knife has to be used. Etherization is required, and this adds to the unpleasantness of the process.

2. For small beginning epitheliomata, those that are no larger than a large pea or a bean, there is no more eligible mode of treatment than by the electrolytic needle. The method appeals to the patient; removal by electricity seems desirous to him. There is but little pain connected with it—anæsthesia is not necessary.

A galvanic battery of at least 20 cells, a few fine watch-maker's brooches and a needle-holder, are all the instruments that are required. The process is exactly the same as that employed for the electrolytic destruction of the hair papillæ. A rheostat or a milliampremeter may be used, but are not necessary. The amount of burning the patient feels and the amount of local reaction that you see at the needle's end, are quite sufficient indices of the strength of the current. In a general way, with a freshly-filled battery, six or seven cells in the circuit is quite enough.

The needle should be well sharpened on an oilstone before introduction, the amount of pain inflicted depending largely on the condition of its point. The needle-holder is attached to the negative pole of the battery, whilst the patient completes the circuit with a sponge electrode attached to the positive pole, at the word of command.

It is important that no mistake be made between the battery poles. If perchance the needle should be attached to the positive pole, and the circuit completed, there would be no destruction of tissue around the needle point, but an oxidation of the needle and the deposition of black particles of oxide of iron in the tissues—a tattooing. It is always easy, however, to distinguish the poles. Immerse the metallic ends of the conducting cords in a glass of water; the end upon which the bubbles of gas form is the negative—the decomposing pole.

The patient holds the handle of the positive electrode in one hand, ready when the word is given to complete the circuit by applying the sponge to the palm of the other hand. The needle is now inserted into the skin just outside the base of the tumor, and passed obliquely through it. The word is given and the current is completed. A burning is felt by the patient at the site of the needle, and minute bubbles of gas appear at the point where it penetrates the skin. The current is allowed to act for a minute or two ; the word is given, and the patient breaks the circuit by withdrawing the positive electrode from the palm of his hand. Then, and not till then, the needle is withdrawn. It is then inserted at the opposite end of the tumor, and the process is repeated. This transfixion is done from all sides, taking care each time to introduce the needle into apparently healthy tissue just outside the evident limits of the growth. Finally, as the blood supply becomes entirely cut off, the tumor assumes a yellowish appearance ; it is filled with gas from the decomposed tissue, and crackles when touched. The entire mass is dead. In a day or two it turns black and is cast off.

No method is more convenient and less painful than this for the destruction of epitheliomatous tumors of small size. The only point needing attention is to exercise care in including a sufficiently large area around the tumor in the destructive process to include all outlying epitheliomatous process.

3. The curette alone is rarely sufficient to complete the cure of an epithelioma. Larger masses of cancerous tissue can be rapidly removed by it, and with a minimum of pain. But it is impossible, save in the cure of a very small growth, to clear out all the rancified epithelial process by this means.

Instruments of various size, known as dermal curettes, are to be employed. Those with large and solid ebony handles, roughened to permit of firm grasping, are much to be preferred to the so-called aseptic instruments with flat steel handles ; since these latter are unhandy to use and liable to slip. Three sizes are required, the largest to rapidly remove masses of tissue, the small ones to go down with into the deeper parts of the growth. The cutting edges of the curettes should be smooth and well-sharpened.

Local anæsthesia is almost always sufficient. Cocaine applied externally, or injected into the periphery of the mass in various places, will do. But a local anæsthetic composed of menthol, 1 part ; chloroform, 10 parts ; sulphuric ether, 15 parts, has given even better results in my hands.

The patient's head is now steadied by an assistant, a mass of absorbent cotton is held with the left hand just under the site of operation. With rapid motion and considerable pressure, the soft, new growth is scraped away. There need be no fear of going too deep. Healthy tissue offers so much greater resistance to this as "prattage," that there is no difficulty in deciding when we come to them.

It is useless to stop after the operation has once begun to apply more of the anæsthetic. These tumors are frequently very vascular, and the blood washes away the remedy. The curetting should be rapidly and thoroughly done when once commenced.

As I have said above, the method is good in its way, but is insufficient alone. There is no more certainty than there is with the knife, that the entire mass has been removed. It must rapidly and thoroughly remove large fungating masses; but it is my invariable rule to follow it up by the fourth method, to be now described.

4. *Escharotics.* These remedies for cancers of the skin have long lain under a cloud undeservedly. The advances of modern surgery have cast into obscurity methods

[TO BE CONTINUED]

